# Sudden infant death syndrome in Brazil: fact or fancy?

**DOI:** 10.1590/S1516-31802008000100009

**Published:** 2008-01-03

**Authors:** Francesca Maia Woida, Fabiano Pinto Saggioro, Maria Alice Rossato Ferro, Luiz Cesar Peres

**Keywords:** Sudden infant death, Infant mortality, Autopsy, Incidence, Diagnosis, Morte súbita do lactente, Mortalidade infantil, Autopsia, Incidência, Diagnóstico

## Abstract

**CONTEXT AND OBJECTIVE:**

The true incidence of sudden infant death syndrome (SIDS) in Brazil is unknown. The aim here was to identify SIDS cases in the city of Ribeirão Preto, State of São Paulo, between 2000 and 2005, in order to estimate its incidence.

**DESIGN AND SETTING:**

Retrospective analysis of data on live births and infant deaths in Ribeirão Preto and from autopsies of infants performed at the Death Verification Service of the Interior (SVOI) between 2000 and 2005.

**RESULTS:**

There were 47,356 live births and 537 deaths, with infant mortality rates ranging from 12.9‰ to 10.9‰ of live births. Among the 24 infants who died possibly due to SIDS and who were autopsied at the SVOI, six were from families living in the municipality (0.13‰ of live births): three (50%) were diagnosed as SIDS, and one each (16.66%) as indeterminate cause, bronchoaspiration and cerebral edema. Two deaths occurred in the first month of life (33.33%) and one each (16.66%) at two, four, six and eight months. Two deaths each (33.33%) occurred in the months of February and December, one each in August and October (16.66%). Four cases (66.7%) occurred in the summer and one each (16.66%) in winter and spring. There was 5:1 predominance of males over females.

**CONCLUSIONS:**

The frequency of SIDS was lower than what has been reported worldwide and in the Brazilian literature, thus suggesting underdiagnosis, indicating the lack of any specific postmortem protocol for SIDS identification and showing the need to implement this.

## RESUMO

**CONTEXTO E OBJETIVO:**

A real incidência da síndrome da morte súbita na infância (SIDS) no Brasil é desconhecida. O objetivo foi identificar casos de SIDS na cidade de Ribeirão Preto, SP, entre os anos de 2000 e 2005 para estimar a sua incidência.

**TIPO DE ESTUDO E LOCAL:**

Análise retrospectiva dos dados relativos aos nascidos vivos e óbitos em Ribeirão Preto, além das necropsias de menores de um ano de idade realizados no Serviço de Verificação de Óbito do Interior (SVOI) entre 2000 e 2005.

**RESULTADOS:**

Houve 47.356 nascidos vivos e 537 óbitos de menores de um ano de idade, com mortalidade infantil variando de 12,9‰ a 10,9‰ nascidos vivos e pós-neonatal de 5,0% a 3,8‰. Dos 24 óbitos possivelmente devidos a SIDS autopsiados no SVOI, 6 eram do município (0,13‰ nascidos vivos), 3 (50%) diagnosticados como SIDS, 1 (16,66%) cada como causa indeterminada, broncoaspiração e edema cerebral. Dois óbitos ocorreram no primeiro mês de vida (33,33%) e um óbito (16,66%) com dois, quatro, seis e oito meses. Dois óbitos (33,33%) ocorreram nos meses de fevereiro e dezembro cada, um caso em agosto e outro em outubro (16,66%). Foram quatro casos (66,7%) no verão, um caso (16,66%) cada no inverno e na primavera. Houve predomínio do gênero masculino sobre o feminino numa proporção de 5:1.

**CONCLUSÕES:**

A freqüência de síndrome da morte súbita na infância foi inferior à da literatura mundial e brasileira, sugerindo subdiagnóstico, falta de protocolo de investigação *post mortem* específico e necessidade de sua implantação.

## INTRODUCTION

Sudden infant death syndrome (SIDS) was first defined by Beckwith et al. in 1970.^1^ It consists of the death of an infant younger than one year of age occurring unexpectedly on the basis of the infant's history and without detecting any adequate cause after postmortem examination, review of the history and examination of the death scene.^2,3^ By definition, there is no cause for this death but, rather, only risk factors. The most important of these is a sleeping position in ventral decubitus (prone position),^4,5^ as extensively demonstrated by the reduction in SIDS incidence that has been achieved by simply switching the sleeping position to dorsal decubitus in all countries in which this procedure has been adopted.^6-11^ Other risk factors are its higher incidence in winter, peak incidence between two and four months of age, higher incidence among infants living in an environment where people smoke, and more common occurrence among preterm babies.^12^

Although SIDS can occur throughout the first year of life, most cases occur between two and four months of life and 95% of these babies are less than six months old when stricken.^12^ The incidence of SIDS varies between different countries, but is usually less than 10 per 1000 live births in industrialized countries, with a rate of 0.3 in Canada and 0.6 in the United States,^13^ although it still represents the most frequent postneonatal cause of death.

The data available for Latin America come from Chile, Argentina and Uruguay, and the last of these countries possibly has the most complete and adequate data.^14^ However, in Argentina, the SIDS rate is available only for the region of Buenos Aires for the year 1995, with a rate of 1.4 per thousand live births.^15^ Regarding Brazil, very few studies are available,^16-20^ with no estimate of the real incidence of this problem, which is considerably underdiagnosed in our country even in places where infant mortality is less than 10 per 100 live births and the health system is adequately established and functioning. A recent epidemiological study in Passo Fundo, State of Rio Grande do Sul, has shown that the possible incidence of SIDS in that town is 1.75 per 1000 live births. It seems that the difficulty in diagnosing these cases is not due to lack of knowledge of SIDS among health professionals, especially pediatricians, but rather to the diffuse belief that there is always a cause of death even though it is not so easy to accept it. Thus, it is common to interpret signs and symptoms of infection as being responsible for death even when they are not of sufficient intensity to explain it. In addition, even pathologists who perform autopsies on these cases tend to identify causes that are only secondary, agonic, events such as bronchoaspiration of the gastric content (curdled milk) among this age group.^17^

The belief that SIDS really exists can only become disseminated when sufficient epidemiological data are obtained, and this will depend on correct diagnosis by all professionals who deal with these deaths. It will be the pediatrician's responsibility to ask for an autopsy and to provide adequate information about clinical findings and doubts, and the pathologists involved in these examinations should use investigation protocols like those that are well established in other places.

## OBJECTIVES

The objective of the present study was to identify the possible cases of SIDS that occurred in the city of Ribeirão Preto, State of São Paulo, Brazil, between 2000 and 2005, and to compare them with existing data in order to determine the probable incidence of SIDS in our environment.

## MATERIAL AND METHODS

We collected all the data on live births and deaths of infants younger than one year of age in the city of Ribeirão Preto from 2000 to 2005, which were available from the Municipal Health Department of Ribeirão Preto. In addition, we obtained all the data on autopsies conducted on infants less than one year old that were performed at the Death Verification Service of the Interior (SVOI) over the same period. The information on the infant mortality rate for this municipality over the study period and data on these infants’ ages at the time of death and their gender (also collected at the same time) were recorded on spreadsheets for analysis, together with the diagnosis declared on the death certificate and information about whether or not an autopsy was performed. We also compared the origin of the diagnosis of SIDS, when present, with the result from the postmortem investigation.

## RESULTS

The infant mortality rate in the municipality of Ribeirão Preto showed a gradual reduction over the study period from 12.9 per 1000 live births in 2000 to 10.9 per 1000 live births in 2005. Concomitantly, postnatal mortality (which includes SIDS cases) went down from 5.0 to 3.8 per 1000 live births (Table 1).

**Table 1.** Epidemiological data relating to births and deaths in different age ranges of subjects living in the municipality of Ribeirão Preto, State of São Paulo, between 2000 and 2005

Year	No. of live births	No. of early neonatal deaths (deaths per 1000 live births)	No. of late neonatal deaths (deaths per 1000 live births)	No. of postneonatal deaths (deaths per 1000 live births)	No. of deaths among children younger than one year of age	Infant mortality rate (per 1000 live births)
2000	8373	56 (6.7)	15 (0.2)	37 (4.4)	108	12.9
2001	8011	50 (6.2)	22 (2.7)	28 (3.5)	100	12.5
2002	7897	43 (5.4)	13 (1.6)	31 (3.9)	87	11.0
2003	7652	38 (5.0)	11 (1.4)	28 (3.7)	77	10.1
2004	7832	50 (6.4)	11 (1.4)	21 (2.7)	82	10.5
2005	7591	49 (6.5)	15 (2.0)	19 (2.5)	83	10.9
**Total**	**47356**	**286 (9.8)**	**87 (1.8)**	**164 (3.5)**	**537 (100)**	

During the study period, there were 537 deaths of infants younger than one year of age, with an overall annual mean of 89.5, ranging from 108 in 2000 to 83 in 2005. Of these, 373 (69.5%) were neonatal deaths (286 early neonatal deaths and 87 late neonatal deaths) and 164 (30.5%) were postnatal deaths. Sixty-two deaths (11.5%) occurred between two and four months of age and 83 (15.5%) between two and six months.

Over the same period, 47,356 live births were recorded, with an overall annual mean of 7892.6 and a range from 7591 in 2005 to 8373 in 2000. There was no difference in the number of deaths per month or per season of the year.

Twenty-four deaths possibly due to SIDS occurred during the period, which were followed by autopsy at the SVOI. Of these, only six involved people living in the municipality, with an incidence of 0.13 cases per 1000 live births. Regarding diagnosis, the cause most frequently recorded on the death certificate was SIDS, with three cases (50%), followed by indeterminate causes, bronchoaspiration of gastric content and cerebral edema in one case each (16.66%). The distribution of these cases by age range showed that two deaths occurred during the first month of life (33.33%) and one death each (16.66%) at two, four, six and eight months, with no case recorded in the remaining months. The months of February and December had the highest number of deaths, with two cases (33.33%) each, followed by the months of August and October, with one case each (16.66%). Regarding the seasons of the year, there were four cases (66.7%) in the summer and one case each (16.66%) in the winter and spring, with no cases recorded in the autumn. All the infants who died were autopsied at the SVOI. There was a difference regarding gender, with predominance of males (five cases, 83.33%) over females (one case, 16.66%), giving a ratio of 5:1 (Table 2).

**Table 2.** Characteristics of the cases of death possibly due to sudden infant death syndrome (SIDS) that were autopsied at the Death Verification Service of the Interior (SVOI) between 2000 and 2005

Case no.	Diagnosis on the death certificate	Age (months)	Gender	Month of death	Season of the year
1	Bronchoaspiration	2	Male	August	Winter
2	SIDS	6	Male	December	Summer
3	Cerebral edema	4	Male	February	Summer
4	Indeterminate cause	1	Male	October	Spring
5	SIDS	1	Male	December	Summer
6	SIDS	8	Female	February	Summer

## DISCUSSION

SIDS is a condition recognized throughout the industrialized world as the main postneonatal cause of death during the first year of life, but it has been little identified in developing countries such as Brazil, even though it is known that when infant mortality is reduced to 10 deaths or less per 1000 live births, the chances of identifying this becomes increasingly high. However, this has not been observed through the reports on infant mortality, even in places with a good health system and death registration system such as the municipality of Ribeirão Preto. Because of the simultaneous functioning of autopsy services of both academic interest, like those performed at the University Hospital of the Faculdade de Medicina de Ribeirão Preto (FMRP), Universidade de São Paulo (USP), and of legal interest, like those performed at the SVOI and at the Legal Medicine Institute, about 36.5% of the deaths occurring in this municipality are autopsied (http://www.saude.ribeiraopreto.sp.gov.br/ssaude/). This increases the reliability of the diagnoses of underlying causes and the final cause of death.

Based on these data, the mortality rate due to SIDS of 0.13 per 1000 live births in Ribeirão Preto is very low, and lower than what has been observed in industrialized countries.^13^ It is also much lower than what was reported by Geib and Nunes^20^, who detected a rate of 1.51 per 1000 live births in Passo Fundo, State of Rio Grande do Sul, in an epidemiological study. The low incidence in Ribeirão Preto possibly indicates underdiagnosis, which would directly influence the parameters studied. Even so, we noted some interesting findings in our results, despite the small absolute number of cases.

In our series, we observed a sharp variation in relation to the months of the year, with an absolute predominance of cases in the summer, in contrast to what is observed in industrialized countries of temperate climate, where there is usually predominance in the winter months,^21^ as first reported by Templeman in 1892.^22^ The question that arises is whether this finding could indicate differences in the risk factors for SIDS in tropical regions, or whether it is simply the influence of environmental factors on cases of death that could be explained if the investigation were more rigorous. Elevated room temperatures may result in a greater loss of water, thus altering the electrolyte concentrations or even resulting in reduced mucus secretion in the respiratory tract. These would increase the possibility of respiratory infections and disorders, and these questions open up perspectives for future investigations.

Most of the cases were observed during the first month of life, with 71.4% occurring up to four months of age and 85.7% up to six months. The literature shows a peak between two and four months of age, with 85% of the cases occurring in the first four months and 95% up to six months.^12^ Although SIDS can occur in the first month of life, such deaths are more likely to be related to perinatal problems than to SIDS and, for this reason, we excluded infants younger than one month of age from our analysis. In any case, this difference between the present data and the data published in other parts of the world needs to be better investigated in order to determine whether or not it really exists, which might directly affect the identification of risk factors.

Another difference observed was in relation to gender, with a clear predominance of male over female gender at a ratio of 5:1. The generally larger number of male victims of SIDS has been noted in several studies, although at a much lower ratio of about 1.5:1.^23^ Once again, the difference may not be real, thereby indicating the need for studies based on stricter criteria for diagnosing SIDS. This is also clear when we consider that 50% of the cases received the diagnosis of SIDS after autopsy, while the remaining diagnoses were death of an undetermined cause, bronchoaspiration of gastric content and cerebral edema of unidentified cause.

The diagnosis of SIDS depends on a detailed and well-documented postmortem examination that includes not only macroscopic and microscopic morphological data, but also microbiological, serological, biochemical, toxicological, radiographic and metabolic data, which are difficult to obtain without an adequate infrastructure. Furthermore, by definition, it is necessary to examine the death scene in order to exclude possible unnoticed environmental causes that might explain the death.^3,24^ In fact, the cases identified as SIDS in our environment did not fulfill these criteria since autopsies in general are limited to macro and microscopic examination of the organs, although not in a systematized manner. The identification of bronchoaspiration of milk as the cause of death in 16.66% of present cases reflected a lack of knowledge on the part of the pathologist performing the autopsy that this is usually an agonic event or an event occurring after death that does not really represent the cause of death.^25^ This had already been pointed out by Peres in 1998 when analyzing possible SIDS cases in the same service (SVOI), in which 80% of such cases were receiving a diagnosis of bronchoaspiration that was not confirmed by histological examination. Although the necroscopic investigation performed in the SVOI is not rigorously adequate, the findings are still relevant since what is expected is that, in the absence of such a detailed investigation, the number of false-positive results should increase and not decrease, i.e. the opposite of what was observed. Furthermore, the remaining studies on SIDS conducted in Brazil did not include necroscopic investigation of all cases, nor did they use internationally accepted criteria.^16,18,19^ This indicates the need for an effort to promote awareness among the medical community, especially pediatricians and pathologists, in order to turn this inadequate situation round.

## CONCLUSIONS

As demonstrated in the present study, the low incidence of SIDS even in a place with an adequate postmortem investigation system is probably due to underdiagnosis. Certain measures should be taken before its real incidence in Brazil can be discussed: a) professionals who deal directly with infants or who work in emergency services will have to be aware that all infants whose death shows the possibility of being SIDS should be subjected to autopsy accompanied by provision of complete and pertinent information, in addition to the clinical suspicions;^26^ b) the services that perform autopsies of this type should institute specific and complete investigation protocols based on the international literature^25^ and promote refreshing of their professionals’ skills, so that the examinations can be made adequate and sufficient for a diagnosis of SIDS. Only after these measures become part of standard practices will we be able to effectively determine what SIDS represents in terms of infant mortality and then be able to carry out studies to analyze the characteristics of this syndrome, such as risk factors and other relevant epidemiological data.

The response to the question indicated in the title, however, is ambiguous: although classic and real cases of SIDS probably exist in Brazil, we are at present experiencing a situation of fantasy in terms of their diagnosis.

## References

[1] Bergman AB, Beckwith JB, Ray CG (1970). Sudden infant death syndrome: proceedings of the second international conference on causes of sudden death in infants.

[2] Zylke JW (1989). Sudden infant death syndrome: resurgent research offers hope. JAMA.

[3] Willinger M, James LS, Catz C (1991). Defining the sudden infant death syndrome (SIDS): deliberations of an expert panel convened by the National Institute of Child Health and Human Development. Pediatr Pathol.

[4] Fleming PJ, Gilbert R, Azaz Y (1990). Interaction between bedding and sleeping position in the sudden infant death syndrome: a population based case-control study. BMJ.

[5] Wigfield RE, Fleming PJ, Berry PJ, Rudd PT, Golding J (1992). Can the fall in Avon's sudden infant death rate be explained by changes in sleeping position?. BMJ.

[6] Markestad T, Skadberg B, Hordvik E, Morild I, Irgens LM (1995). Sleeping position and sudden infant death syndrome (SIDS): effect of an intervention programme to avoid prone sleeping. Acta Paediatr.

[7] Engelberts AC, de Jonge GA, Kostense PJ (1991). An analysis of trends in the incidence of sudden infant death in The Netherlands 1969-89. J Paediatr Child Health.

[8] Spiers PS, Guntheroth WG (1994). Recommendations to avoid the prone sleeping position and recent statistics for sudden infant death syndrome in the United States. Arch Pediatr Adolesc Med.

[9] Beal SM (1993). Sudden infant death syndrome (SIDS) in South Australia related to sleeping conditions. Med J Aust.

[10] Waters KA, Gonzalez A, Jean C, Morielli A, Brouillette RT (1996). Face-straight-down and face-near-straight-down positions in healthy, prone-sleeping infants. J Pediatr.

[11] Dwyer T, Ponsonby AL (1996). The decline of SIDS: a success story for epidemiology. Epidemiology.

[12] Valdes DaPena M, Naeye RL, Gilbert Barness E, Gilbert Barness EF (1997). Sudden infant death syndrome. Potter's pathology of the fetus and infant.

[13] Hunt CE, Hauck FR (2006). Sudden infant death syndrome. CMAJ.

[14] Landi K, Gutierrez C, Sampson B (2005). Investigation of the sudden death of infants: a multicenter analysis. Pediatr Dev Pathol.

[15] Nelson EA, Taylor BJ (2001). International Child Care Practices Study: infant sleep position and parental smoking. Early Hum Dev.

[16] Victora CG, Nobre LC, Lombardi C (1987). Quadro epidemiológico das mortes súbitas na infância em cidades gaúchas (Brasil). [Epidemiologic picture of sudden infant death in cities of Rio Grande do Sul (Brazil)]. Rev Saude Publica.

[17] Peres LC (1998). Sudden unexpected infant death syndrome in Ribeirão Preto, Brazil. Sao Paulo Med J.

[18] Nunes ML, Martins MP, Nelson EA, Cowan S, Cafferata ML, Costa JC (2002). Orientações adotadas nas maternidades dos hospitais-escola do Brasil, sobre a posição de dormir. [Instructions from teaching hospital maternity wards to parents concerning the sleeping position of newborns]. Cad Saúde Pública = Rep Public Health.

[19] Geib LTC, Nunes ML (2006). Hábitos de sono relacionados à síndrome da morte súbita do lactente: estudo populacional. [Sleeping habits related to sudden infant death syndrome: a population-based study]. Cad Saúde Pública = Rep Public Health.

[20] Geib LTC, Nunes ML (2006). The incidence of sudden death syndrome in a cohort of infants. J Pediatr (Rio J).

[21] Beal S, Porter C (1991). Sudden infant death syndrome related to climate. Acta Paediatr Scand.

[22] Templeman C (1892). Two hundred and fifty-eight cases of suffocation of infants. Med Chir Soc (Edinburgh).

[23] Brooke H, Gibson A, Tappin D, Brown H (1997). Case-control study of sudden infant death syndrome in Scotland, 1992-5. BMJ.

[24] Krous HF, Beckwith JB, Byard RW (2004). Sudden infant death syndrome and unclassified sudden infant deaths: a definitional and diagnostic approach. Pediatrics.

[25] Centers for Disease Control and Prevention (CDC) (1996). Guidelines for death scene investigation of sudden, unexplained infant deaths: recommendations of the interagency panel on sudden infant death synd. MMWR Recomm Rep.

[26] Peres LC (2005). SIDS-Síndrome infantil da defunção súbita. [SIDS-Sudden infant death syndrome]. Medicina (Ribeirão Preto).

